# Cortisol-Related Signatures of Stress in the Fish Microbiome

**DOI:** 10.3389/fmicb.2020.01621

**Published:** 2020-07-14

**Authors:** Tamsyn M. Uren Webster, Deiene Rodriguez-Barreto, Sofia Consuegra, Carlos Garcia de Leaniz

**Affiliations:** Centre for Sustainable Aquatic Research, College of Science, Swansea University, Swansea, United Kingdom

**Keywords:** stress response, microbiota, glucocorticoid, *Salmo salar*, lactic acid bacteria

## Abstract

Exposure to environmental stressors can compromise fish health and fitness. Little is known about how stress-induced microbiome disruption may contribute to these adverse health effects, including how cortisol influences fish microbial communities. We exposed juvenile Atlantic salmon to a mild confinement stressor for two weeks. We then measured cortisol in the plasma, skin-mucus, and feces, and characterized the skin and fecal microbiome. Fecal and skin cortisol concentrations increased in fish exposed to confinement stress, and were positively correlated with plasma cortisol. Elevated fecal cortisol was associated with pronounced changes in the diversity and structure of the fecal microbiome. In particular, we identified a marked decline in the lactic acid bacteria *Carnobacterium* sp. and an increase in the abundance of operational taxonomic units within the classes Clostridia and Gammaproteobacteria. In contrast, cortisol concentrations in skin-mucus were lower than in the feces, and were not related to any detectable changes in the skin microbiome. Our results demonstrate that stressor-induced cortisol production is associated with disruption of the gut microbiome, which may, in turn, contribute to the adverse effects of stress on fish health. They also highlight the value of using non-invasive fecal samples to monitor stress, including simultaneous determination of cortisol and stress-responsive bacteria.

## Introduction

Stress can be broadly defined as a state in which a series of adaptive responses re-establish homeostasis following exposure to a stressor (Chrousos, 1998; Schreck and Tort, 2016). In fish, the stress response includes activation of the hypothalamus–pituitary–interrenal (HPI) axis, culminating in the release of glucocorticoids from interrenal cells located in the head kidney (Barton, 2002). As for mammals, cortisol is the predominant glucocorticoid released as part of the primary stress response, and is critical for mediating adaptive metabolic, physiological, and behavioral adjustments (Schreck and Tort, 2016). However, prolonged elevation of cortisol, due to extended or repeated exposure to a stressor, is often associated with adverse health effects. In intensive aquaculture, farmed fish are frequently exposed to stressors such as crowding and handling, which can impact health and welfare, and threaten aquaculture sustainability (Conte, 2004). In the wild as well, natural fish populations are increasingly subject to multiple anthropogenic stressors which threaten their sustainability. In particular, stress-mediated impairment of immune function has been widely described in cultured and wild fish, and associated with an increased susceptibility to disease (Miller et al., 2014; Yada and Tort, 2016; Ellison et al., 2018; Uren Webster et al., 2018).

Recent research has revealed the diverse influence of microbiota and their metabolites on many aspects of host health and fitness, including digestion and nutrient uptake, metabolism and immune development (Hooper et al., 2012; Rea et al., 2016). In mammals, stress is well known to disrupt the microbiome which, in turn, has been associated with long term health effects in the host, including metabolic and immune impairment, and a range of diseases (Foster et al., 2017; Tetel et al., 2018). The mechanisms by which stress impacts the microbiome are complex, and not fully understood. Elevated plasma cortisol, resulting from social or psychological stress, has been associated with alterations in the structure and/or diversity of the mammalian microbiome, including the abundance of lactic acid bacteria and opportunistic pathogens (Galley et al., 2014; Jašarević et al., 2015; Mudd et al., 2017). Direct administration of glucocorticoids has also been shown to exert stimulatory and inhibitory effects on specific microbial taxa, together with wider effects on host metabolism in some cases (Jentsch et al., 2013; Huang et al., 2015; Petrosus et al., 2018; Wu et al., 2018). Additionally, while host stress response influences the microbiome, the microbiome can also influence host stress response. Microbiota and their metabolites are known to exert effects throughout the mammalian HPA axis, influencing glucocorticoid synthesis, release and signaling pathways (Simard et al., 2014; Burokas et al., 2017; de Weerth, 2017; Vodička et al., 2018).

Disruption of the microbiome is likely to represent an important mechanism by which stress affects fish health, welfare and performance in aquaculture, as well as in the natural environment. There is some evidence that environmental and social stressors disrupt microbial communities associated with the fish gut and skin (Boutin et al., 2013; Sylvain et al., 2016; Zha et al., 2018) however, a potential role of cortisol in mediating these effects is unknown. Therefore we exposed juvenile Atlantic salmon to a mild, repeated confinement stressor, measured cortisol in plasma, feces and skin, and examined the relationship between cortisol and the gut and skin microbiome. We hypothesized that the extent of change in the fish microbiome might be predicted by individual variation in the magnitude of cortisol stress response.

## Materials and Methods

### Confinement Stress Experiment

Prior to the start of the experiment 8 month post hatch Atlantic salmon fry (mass 3.92 ± 0.11 g; fork length 7.46 ± 0.07 cm), were housed in stock tanks (80 L) supplied with a recirculating flow of aerated and de-chlorinated tap water, with a temperature of 15 ± 0.5°C and photoperiod of 12L:12D. Water oxygen saturation (>90%), ammonia (<0.02 mg/L), nitrite (<0.01 mg/L), nitrate (<15 mg/L), and pH (7.5 ± 0.2) were maintained within the optimal range for the species. Fish were fed with a commercial feed (Skretting Nutra Parr) at 3% body weight/day.

Experimental fish were assigned at random to the control and confinement-stress treatment groups, with three replicate 20 L tanks per group, each containing 28 fish. Confinement consisted of slowly lowering the water volume in each tank (via draining) from 20 to 5 L for 1 h, and this was repeated every day at the same time (1100 h) for two weeks. All other husbandry conditions were as before. At the end of the experiment, fish were euthanized using Phenoxyethanol (0.5 mg/L), followed by destruction of the brain according to UK Home Office regulations. The fish were measured (fork length), weighed (wet weight), and Fulton’s condition factor was calculated as a measure of body condition. Blood samples were collected from the caudal vein using heparinized capillary tubes, centrifuged (5 min, 5000 × *g*) and the plasma supernatant was removed and stored at −80°C prior to cortisol analysis. For each fish, a sample of skin-associated mucus for microbiome analysis was collected by swabbing the left side lateral line five times using Epicentre Catch-All^TM^ Sample Collection Swabs (Cambio, Cambridge, United Kingdom). A sample of skin-associated mucus for cortisol analysis was collected by scraping mucus from a 2 cm^2^ region of skin on the left-hand side of the fish, above the lateral line between the head and dorsal fin, using a scalpel blade. Fecal samples were collected from each fish by gently pressing along the length of the abdomen and collecting expelled feces, which were then split evenly between samples for cortisol and microbiome analysis. All skin-mucus and fecal samples were directly frozen at −80°C prior to analysis.

### Cortisol Measurement

Quantification of cortisol concentration in plasma, skin mucus and fecal samples was performed using the DetectX Cortisol Enzyme Immunoassay Kit (Arbor Assays, Ann Arbor, MI, United States), according to the manufacturer’s recommendations. Briefly, plasma samples were pre-treated with dissociation reagent then diluted in assay buffer (1:50 final dilution) before cortisol measurement. Fecal samples were suspended in 100 μL ethanol, vortexed for 30 min, centrifuged (5 min, 5000 × *g*), then the supernatant was then diluted in assay buffer (1:20) before cortisol measurement. Skin-mucus samples were suspended in 100 μL 1M Tris–HCl, vortexed for 30 min and centrifuged (5 min, 5000 × *g*), then the supernatant was used directly in the assay without dilution.

Cortisol concentration was measured in the plasma, feces and skin-mucus for a total of 60 individual fish (40 stressed fish and 20 controls; distributed evenly amongst replicate tanks). We included a greater number of stressed individuals in the analysis in order to encompass variation in cortisol stress responses [i.e., high and low responders (Pottinger and Carrick, 1999; Samaras et al., 2016)]. Each of these 180 samples was analyzed in duplicate, across five 96-well plates. Cortisol concentration was calculated based on a standard curve run on each plate, and adjusted for dilution factor and initial sample volume (plasma) or weight (skin-mucus/feces). Inter-assay variability, measured as the coefficient of variation (CV%) of four repeats across the five plates, was 4.62% and the lower limit of detection was 76.4 pg/mL. We removed one outlier of fecal cortisol (47.3 ng/g) from the stressed group (most likely resulting from an error in sample preparation) using Tukey’s 1.5^∗^IQR method, as it was 3.4× higher than the mean value (13.76 ng/g) and 1.6× higher than the next highest value in this group (29.1 ng/g).

### 16S rRNA Amplicon Sequencing

16S rRNA amplicon sequencing was performed using the fecal and skin mucus samples for the same 60 individual fish for which cortisol quantification was performed. DNA was extracted from all samples using the PowerSoil DNA Isolation Kit (Qiagen) according to the manufacturer’s instructions, but including an additional 10 min incubation step at 65°C prior to bead beating. Libraries were prepared according to the Illumina Metagenomic Sequencing Library Preparation protocol, targeting the V3–V4 hypervariable region of the universal prokaryote 16S rRNA gene using the primers 341F and 785R. The first PCR reaction consisted of an initial denaturation at 95°C for 3 min, followed by 25 cycles of 95°C for 30 s, 55°C for 30 s, and 72°C for 30 s, then a final elongation at 72°C for 5 min, using 12.5 ng input genomic DNA, 0.2 μM of primers and KAPA HiFi HotStart ReadyMix (Kapa Biosystems) in a total volume of 25 μL. All products were purified with Agencourt Ampure XP beads (Beckman Coulter) then used as template for the second PCR reaction to add indexed sequencing adaptors to each library (Nextera XT Indices, Illumina). The reaction conditions used were the same as before, but using eight cycles, and a total reaction volume of 50 μL. The final libraries were purified using AMPure XP beads, and the presence of amplicons of the expected size was checked using gel electrophoresis. All libraries were quantified using a Qubit 3.0 Fluorometer (Thermo Fisher Scientific), multiplexed in equimolar concentrations and sequenced on an Illumina MiSeq (2x 300 bp).

Raw sequence reads were quality filtered using Trimmomatic (Bolger et al., 2014) before analysis with mothur v1.39 (Schloss et al., 2009). Concatenated reads were aligned to the Silva seed reference database (version 128; Quast et al., 2012) chimeric reads were removed using UCHIME (Edgar et al., 2011) and bacteria and archaea contigs were classified using the Silva reference taxonomy using a minimum bootstrapped confidence score of 80% (Wang et al., 2007). Contigs were clustered into operational taxonomic units (OTUs) using mothur, based on 97% sequence similarity. Reads assigned as chloroplasts, mitochondria and host DNA were filtered out, and singleton OTUs removed from the dataset then all fecal samples were subsampled to an equal depth of 19,724 reads and all skin samples were subsampled to 10,133 reads. Measures of alpha diversity (Chao1 richness and Shannon diversity) for each sample were calculated in mothur.

### Statistical Analysis

All statistical analysis was performed in R (v3.6.1; R Development Core Team and R Core Team, 2019). Firstly, we assessed whether non-invasive measurements of fecal and skin cortisol were indicative of cortisol in blood plasma by calculating the Pearson correlation coefficient. We then employed linear mixed effects models (LMM) using the lme4 package (Bates, 2007) to examine the effects of confinement stress and fish size on cortisol concentration in plasma, skin, and feces, using tank identity as a random factor to account for non-independence. Cortisol data were log transformed to meet model assumptions. We also used LMM to examine the effects of confinement stress, fish size, and measured feces/skin cortisol on faces/skin microbial alpha diversity (Chao1 richness and Shannon diversity), including tank as a random factor. We used fish length as a covariate to control for size effects as it had a lower coefficient of variation (CV = 0.067) than fish mass (CV = 0.216). To achieve model simplification, we started with a model with all main effects and interactions and selected the model with the lowest AIC value via backward selection using the step and drop1 functions and the lmerTest package (Kuznetsova et al., 2017). A minimal adequate model was then refitted via Restricted Maximum Likelihood, or as a linear model when the random component (tank identity) did not improve model fit compared to the fixed effects only model, as indicated by the likelihood ratio test (LRT). We used the VCA package (Schuetzenmeister, 2019) to estimate the amount of variability in cortisol due to tank effects and differences among individual fish.

Comparison of microbial community structures (beta diversity) was performed within the Vegan package (Oksanen et al., 2019) using the Bray–Curtis dissimilarity index. Non-metric multidimensional scaling (NMDS) ordination of Bray–Curtis distances were visualized, including measured cortisol concentration as an environmental vector. Multivariate statistical analysis of microbial community separation in the fecal and skin samples was performed by PERMANOVA using Adonis, with confinement stress and measured fecal/skin cortisol as predictors. Statistical analysis of OTU abundance was performed using DeSeq2 (Love et al., 2014). The effect of confinement stress and fecal/skin cortisol on relative abundance of fecal/skin OTUs was tested using a multifactorial design. Within the DeSeq model, low coverage OTUs were independently filtered to optimize power for identification of differentially abundant OTUs at a threshold of alpha = 0.05. Outlier detection and moderation of OTU level dispersion estimates were performed using default settings, and OTUs were considered significantly differentially abundant at FDR <0.05.

## Results

### Relation Between Plasma Cortisol and Non-invasive Measures of Cortisol in Feces and Skin

**FIGURE 1:**
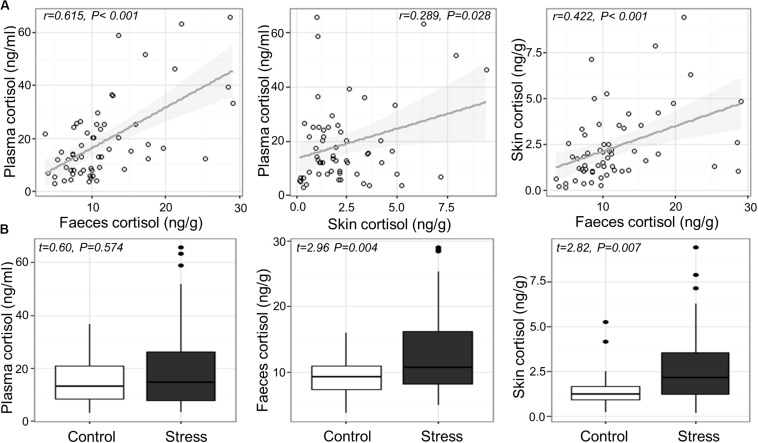
**(A)** Relation between measured cortisol in the plasma, skin and feces across individual fish and **(B)** measured cortisol in the plasma, feces and skin of Atlantic salmon exposed to confinement stress compared to control fish.

Cortisol concentrations ranged from 2.9 to 65.8 ng/mL in blood plasma, 3.6–29.1 ng/g in feces, and 0.14–9.45 ng/g in skin mucus across all samples. Significant positive correlations were found between plasma and fecal cortisol (Pearson’s *r_5__6_* = 0.615, *P* < 0.001), between plasma and skin cortisol (*r_5__6_* = 0.289, *P* = 0.028), and between fecal and skin cortisol (*r_57_* = 0.422, *P* < 0.001; Figure 1A). Variance component analysis indicated that 82–85% of the variation in cortisol was due to variation between individuals, and 0–18% was due to variation between tanks.

### Effects of Confinement Stress on Cortisol

Confinement stress induced a modest elevation in plasma, feces and skin cortisol concentration (fold increases of 1.37, 1.42 and 1.82, respectively). There was a significant increase in cortisol in the feces and skin of stressed fish compared to unstressed controls (Welch two sample *t*-test; feces, *t*_56.766_ = 2.955, *P* = 0.004; skin, *t*_52.917_ = 2.819, *P* = 0.007), but not in blood plasma (LMM stress effect, *t*_4.785_ = 0.603, *P* = 0.574; Figure 1B), which showed a significant tank effect (LRT *χ*^2^ = 6.01, *P* = 0.014). There was no association between cortisol and the length, weight or body condition of fish at the end of the experiment (*P* > 0.2 in all cases).

### Microbiome Analysis

16S rRNA amplicon sequencing generated a total of 10.03 and 4.83 M raw sequence reads across all of the fecal and skin mucous samples, respectively. All sequence data are available from the European Nucleotide Archive under accession PRJEB32276.

After quality filtering, OTU clustering, filtering of non-target sequences and singletons, we obtained a total of 8559 fecal OTUs and 4459 skin OTUs (Supplementary Tables S1, S2). Across all samples, the fecal microbiome was strongly dominated by the phylum Firmicutes, with smaller numbers of Actinobacteria and Proteobacteria, while the most abundant OTUs across all samples were *Carnobacterium* sp. and *Peptostreptococcus* sp. In the skin microbiome, the dominant phyla were Proteobacteria, Actinobacteria, and Bacteroidetes, while the most abundant OTUs were *Janthinobacterium* sp. and *Propionibacterium* sp.

### Association Between Cortisol and Microbial Diversity

**FIGURE 2:**
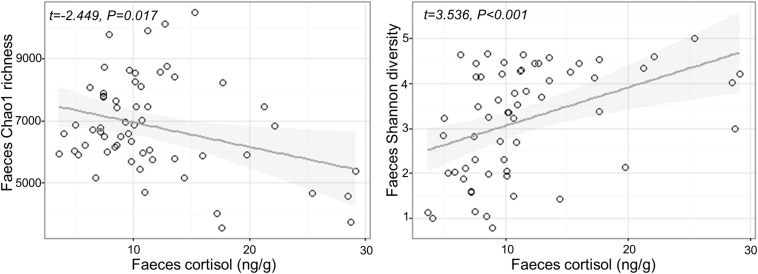
Relationship between measured cortisol and microbial alpha diversity (Chao1 richness and Shannon diversity) in the feces.

Fecal cortisol was negatively correlated with fecal Chao1 microbial richness (Chao1 Cortisol estimate: −79.17 ± 32.33, *t*_1,57_ = −2.449, *P* = 0.017), but positively correlated with Shannon diversity (Shannon Cortisol estimate: 0.08 ± 0.024, *t*_1,5__7_ = 3.536, *P* < 0.001; Figure 2). There was no effect of confinement stress, fish size or tank identity on fecal microbial diversity beyond that accounted by an increase in cortisol (*P* > 0.4 in all cases). For the skin, there was no significant effect of confinement stress, skin cortisol, or fish size on skin microbial Chao1 richness of Shannon diversity (*P* > 0.1 in all cases), although there were significant tank effects for skin Chao1 richness (LRT *χ*^2^ = 15.53, *P* < 0.001).

Microbial community structural diversity was performed based on the Bray-Curtis dissimilarity metric, and visualized using NMDS analysis (Supplementary Figure S1). For the skin microbiome there was no detectable effect of confinement stress or skin cortisol concentration on beta diversity (Stress: *F*_1,53_ = 1.263, *P* = 0.151, Cortisol: *F*_1,53_ = 0.960, *P* = 0.475).

### Association Between Cortisol and Microbial Composition

**FIGURE 3:**
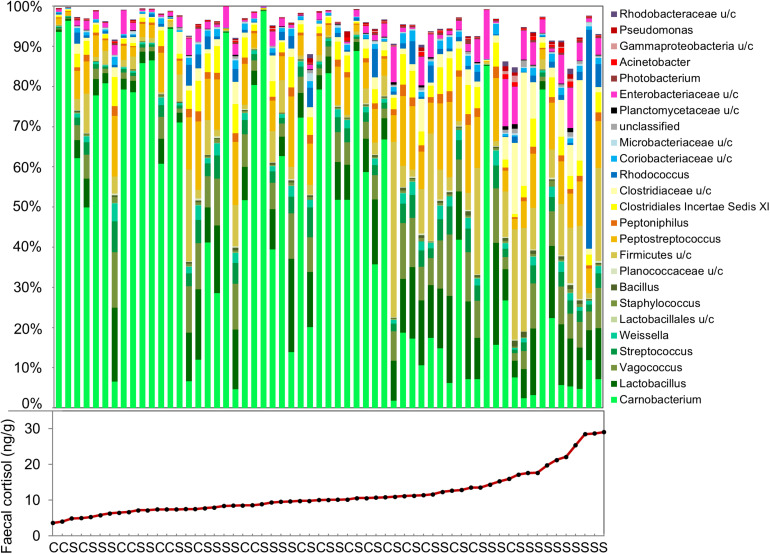
Genus-level composition of the fecal microbiome, and measured fecal cortisol concentrations in individual fish. (C: control, S: confinement stress).

The effects of confinement stress and cortisol concentration on OTU relative abundance was investigated using DeSeq2. For the fecal microbiome, the abundance of 44 OTUs (27 increased and 17 decreased) were significantly associated with fecal cortisol concentration, but only one OTU (*Vagacoccus* sp.) was significantly elevated in the confinement stress group independently of cortisol (Figure 3 and Supplementary Table S3). Strikingly, of the 17 OTUs which were negatively associated with cortisol concentration, the vast majority (15) were classified as belonging to the genus *Carnobacterium* sp. in the order Lactobacillales, including the most abundant OTU overall. Of the OTUs that were positively associated with fecal cortisol concentration, 10 (37%) were from the class Gammaproteobacteria and, notably, two highly abundant OTUs from the family Clostridiaceae were also increased. In contrast, for the skin microbiome, no OTUs were significantly associated with either confinement stress or measured skin cortisol concentration.

## Discussion

Out study shows that an increase in cortisol, resulting from exposure to a confinement stressor, is associated with significant changes in the gut, but not the skin, microbiome of juvenile Atlantic salmon. Individual cortisol stress response was variable and we identified a distinctive relationship between fecal cortisol concentration and the diversity and composition of the feces microbiome, particularly the abundance of the lactic acid bacteria, *Carnobacterium* sp.

Exposure to a reoccurring, low-level aquaculture-relevant stressor (confinement) increased cortisol concentration in the skin and feces of juvenile salmon compared to control fish. There was considerable variation in cortisol response among individuals, which is consistent with the existence of low and high cortisol responders (Pottinger and Carrick, 1999; Samaras et al., 2016). We also identified a positive association between plasma and fecal cortisol, and, to a lesser extent, between plasma and skin-mucus cortisol. Plasma cortisol is typically used to measure the stress response in fish, but blood sampling is invasive and may require terminal sampling in the case of small fish (Sadoul and Geffroy, 2019). Plasma cortisol concentration is also known to be influenced by acute spikes in glucocorticoid production, for example caused by handling prior to sampling, which may mask underlying stress levels (Bertotto et al., 2009). Our results add to that of other recent studies suggesting that fecal and skin sampling provide non-destructive alternatives to measuring plasma cortisol in Atlantic salmon (Bertotto et al., 2009; Lupica and Turner, 2009; De Mercado et al., 2018) which can also be linked directly to microbiome analysis.

Across all fish, including high and low cortisol responders and non-stressed controls, we identified a strong association between measured cortisol in the feces and both alpha and beta measures of gut microbiome diversity. Fecal cortisol was negatively associated with Chao1 richness, but positively associated with Shannon diversity, suggesting that there were fewer, but more evenly distributed, bacterial taxa in stressed fish. This is likely to reflect an inhibitory effect of cortisol on dominant OTUs normally present in non-stressed individuals. In particular, there was a striking decline in *Carnobacterium* sp. with increasing levels of fecal cortisol, including the most abundant OTU in non-stressed fish, together with +10 other OTUs assigned to this genus. *Carnobacterium* (order Lactobacillales, class Bacilli, phylum Firmicutes) is a genus of facultatively anaerobic, cold tolerant lactic acid bacteria comprising +12 species (Pikuta and Hoover, 2014). This genus, particularly *Carnobacterium pisciola, Carnobacterium divergens*, and *Carnobacterium inhibens*, is commonly found in the intestinal communities of healthy fish, including Atlantic salmon (Ringø et al., 2001). *Carnobacterium* sp. are also widely used as probiotics in aquaculture, due to their beneficial effects on gut health and fish performance, and their ability to inhibit the growth of several common fish pathogens (Ringø et al., 2001; Ringø, 2008).

Individuals that displayed a high cortisol response to confinement stress had a distinct fecal microbiome, that was quite different from that of non-responsive fish and from control fish that had low baseline cortisol levels. Alongside a marked decline in *Carnobacterium* sp., this structural change was characterized by a marked increase in the relative abundance of two Clostridiaceae OTUs. This family (class Clostridia, phylum Firmicutes) is commonly found in the gut of mammals and fish, but includes a number of opportunistic pathogens. Although relatively little is known about the role that Clostridiaceae play in the fish gut, in mammals an increased abundance of this family has been associated with microbial dysbiosis, intestinal inflammation and gastrointestinal diseases (Lopetuso et al., 2013; Muñiz Pedrogo et al., 2018). Several OTUs within the class Gammaproteobacteria, including two *Yersinia* sp., *Pseuodomonas* sp., *Acinetobacter* sp., and *Aeromonas* sp., were also particularly abundant in fish that had high levels of fecal cortisol. These genera include a range of opportunistic fish pathogens and tend to increase following exposure to different types of environmental stressors in fish (Austin and Austin, 2007; Boutin et al., 2013), suggesting they may represent a common signature of stress exposure. In mammals, experimental administration of cortisol results in a similar reduction in probiotic lactic acid-producing bacteria and an increase in pro-inflammatory microbiota (Huang et al., 2015; Petrosus et al., 2018; Wu et al., 2018), suggesting that these bacteria taxa could represent useful biomarkers of stress across vertebrates.

Our results demonstrate how an increase in cortisol can predict changes in the diversity and structure of the salmon gut microbiome, which may in turn contribute to the adverse effects of stress on fish health. This is consistent with results of previous studies that experimentally administered glucocorticoids to rats, mice, and pigs (Huang et al., 2015; Petrosus et al., 2018; Wu et al., 2018). However, it is not clear exactly how cortisol may affect different taxa within complex host-associated microbial communities. Potential inhibitory mechanisms could include direct toxicity, metabolic impairment, disruption of ion-regulation, endocrine signaling or nutrient depletion, similar to the effects of other chemical or physical stressors (Weber et al., 2014; Harms et al., 2016). At the same time, taxa more tolerant of cortisol may flourish in the absence of previous niche completion (Hibbing et al., 2009) and cortisol is also known to specifically promote the growth of certain oral pathogens *in vitro* (Jentsch et al., 2013). However, the overall relationship between stress response, cortisol and the microbiome is complex. The microbiome may be influenced by other stress hormones, by interactions amongst microbes or with the host immune system, and microbiota and/or their metabolites can also influence host stress response signaling (Burokas et al., 2017; de Weerth, 2017).

The relationship between cortisol and the microbiome is also likely to depend on the nature of the microbial community, and the stress response. In contrast to the fecal microbiome, we found no significant effects of confinement stress or skin cortisol concentration on the skin microbiome. It is possible that skin-associated communities, which are dominated by Proteobacteria with much lower levels of Lactobacilliales, are less sensitive to cortisol than fecal microbiota. On the other hand, cortisol concentrations in the skin mucus were much lower than those measured in fecal samples, which may also help explain the lack of observed effects of skin cortisol on the skin microbiome.

To conclude, our study shows that an increase in the concentration of fecal cortisol after exposure to a mild confinement stressor is associated with changes in the diversity and composition of the intestinal microbiome. Notably, these included reduced abundance of *Carnobacterium*, a lactic acid bacteria commonly used as a probiotic in aquaculture, and increased levels of several genera containing pro-inflammatory and opportunistic bacterial pathogens. Given the fundamental influence of microbiota and their metabolites on many aspects of host health, this suggests that disruption of the gut microbiome is likely to contribute to the adverse effects of stress on immune function and disease resistance. These results have important implications for health and welfare of fish exposed to environmental stressors, and more broadly, to research on stress-related diseases, such as metabolic syndrome, obesity, and IBD, which have been associated with microbiome dysbiosis. Finally, our study demonstrates that both cortisol measurements and microbiome analysis can be performed simultaneously on fecal and skin samples collected non-invasively, which could represent a valuable screening tool for evaluating stress in fish.

## Author’s Note

This manuscript has been released as a pre-print at bioRxiv, Uren Webster et al. (2019).

## Data Availability Statement

The datasets generated for this study can be found in the European Nucleotide Archive (accession PRJEB32276).

## Ethics Statement

The animal study was reviewed and approved by Swansea Animal Welfare and Ethical Review Body, Swansea University (number IP-1415-2).

## Author Contributions

TU and DR-B performed the experiment. TU and CG analyzed the data. TU drafted the manuscript. All authors designed the study and contributed to the final version of the manuscript.

## Conflict of Interest

The authors declare that the research was conducted in the absence of any commercial or financial relationships that could be construed as a potential conflict of interest.
