# Supplementary material for: Cortisol-Related Signatures of Stress in the Fish Microbiome
Source: Front Microbiol. 2020 Jul 14;11:1621. doi: 10.3389/fmicb.2020.01621 (PMC7381252; doi:10.3389/fmicb.2020.01621)
Supplement: Supplementary file 1 [file Data_Sheet_1.PDF]

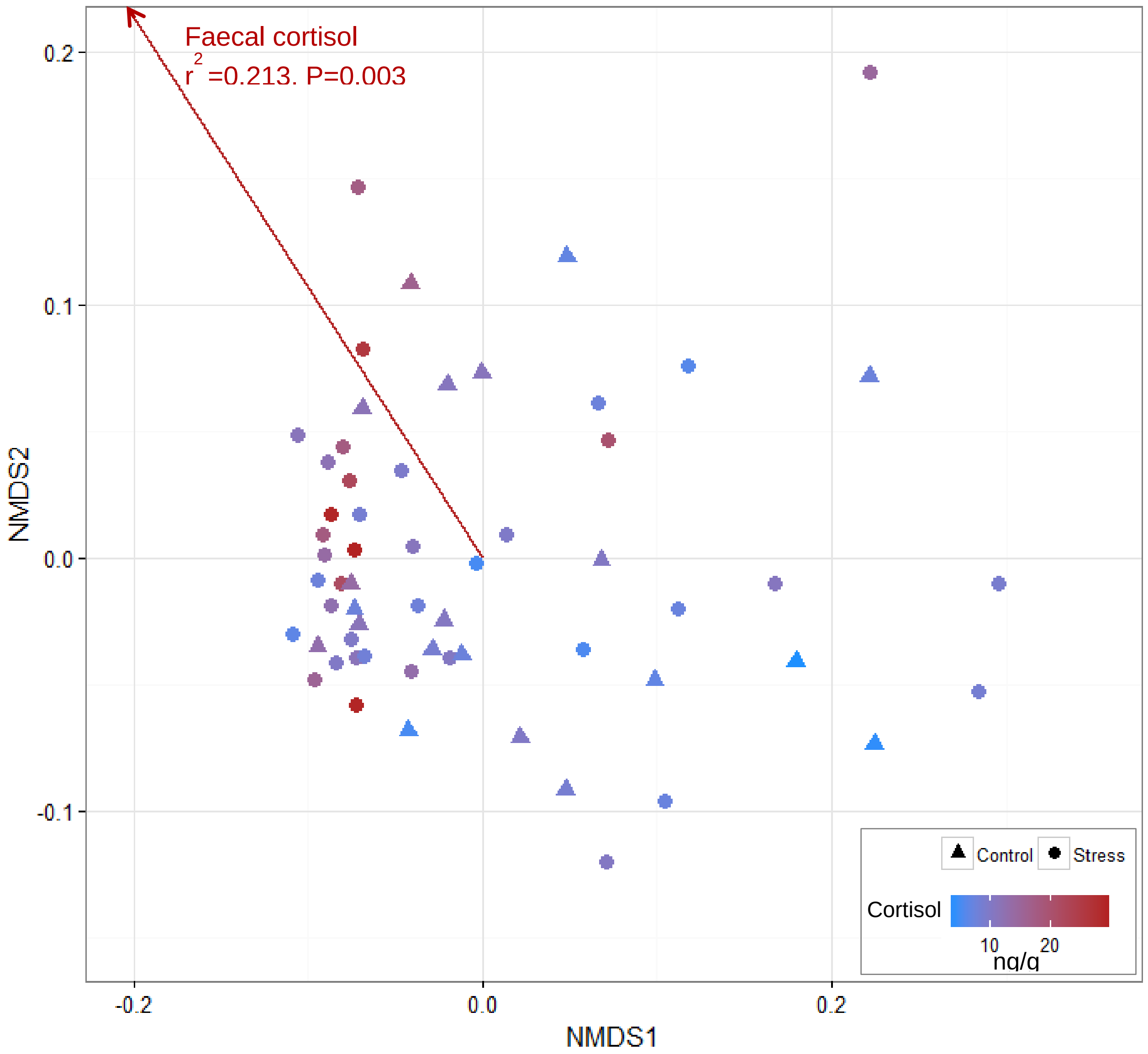

Figure S1. Structural analysis of the faecal microbiome. Non-metric multidimensional scaling ordination was performed based on the Bray-Curtis dissimilarity index, and correlated with measured faecal cortisol concentration. Individual points are colour-coded based on cortisol concentration.
