# Supplementary material for: Cortisol-Related Signatures of Stress in the Fish Microbiome
Source: Front Microbiol. 2020 Jul 14;11:1621. doi: 10.3389/fmicb.2020.01621 (PMC7381252; doi:10.3389/fmicb.2020.01621)
Supplement: Supplementary file 5 [file Data_Sheet_5.PDF]

Table S4. Morphological and cortisol measurements for all fish.

Fish ID	Tank	Treatment	Faeces cortisol (ng/g)	Skin cortisol (ng/g)	Plasma cortisol (ng/ml)	Weight (g)	Fork length (cm)	Condition factor
53	Control A	Control	3.65	0.63	21.50	5.35	8.2	0.97
51	Control A	Control	4.06	0.21	6.66	3.66	7.4	0.90
21	Control B	Control	5.00	0.37	2.97	5.96	8.0	1.16
26	Control B	Control	6.55	1.73	8.86	4.15	7.4	1.02
82	Control C	Control	6.73	1.19	11.99	5.58	8.5	0.91
54	Control A	Control	7.44	0.33	6.31	4.33	7.9	0.88
30	Control B	Control	7.45	1.03	14.08	4.99	7.9	1.01
28	Control B	Control	7.55	1.36	24.14	4.60	7.8	0.97
59	Control A	Control	8.57	1.04	13.06	3.27	7.1	0.91
58	Control A	Control	8.61	0.97	17.28	3.94	7.8	0.83
57	Control A	Control	9.82	0.65	8.75	3.77	7.4	0.93
83	Control C	Control	10.06	1.52	7.91	4.84	8.2	0.88
56	Control A	Control	10.23	1.3	20.36	3.48	7.3	0.89
90	Control C	Control	10.62	5.24	3.76	3.88	7.5	0.92
86	Control C	Control	10.86	2.47	12.98	6.23	8.5	1.01
25	Control B	Control	11.20	2.18	23.53	5.40	8.6	0.85
87	Control C	Control	11.43	1.27	NA	3.60	7.0	1.05
27	Control B	Control	12.69	1.05	36.65	3.86	8.0	0.75
84	Control C	Control	13.56	4.17	20.11	3.43	7.2	0.92
88	Control C	Control	15.97	1.6	25.22	3.21	7.0	0.93
32	stress B	Stress	4.92	0.14	5.38	6.02	8.5	0.98
9	stress A	Stress	5.29	2.53	11.99	6.54	8.9	0.93
38	stress B	Stress	5.86	1.83	14.69	4.27	7.9	0.87
61	stress C	Stress	6.30	0.48	3.96	3.85	7.6	0.88
62	stress C	Stress	7.18	1.11	8.00	4.83	7.8	1.02
6	stress A	Stress	7.20	3.33	4.45	4.01	7.7	0.88
63	stress C	Stress	7.45	1.81	13.64	7.39	9.0	1.01
10	stress A	Stress	7.55	3.02	7.91	4.90	8.0	0.96
7	stress A	Stress	7.76	1.14	12.16	5.17	8.3	0.90
68	stress C	Stress	7.95	2.03	25.81	3.37	7.1	0.94
34	stress B	Stress	8.43	0.38	26.52	4.34	7.7	0.95
8	stress A	Stress	8.50	7.14	6.77	3.46	7.5	0.82
66	stress C	Stress	8.89	4.98	7.90	4.58	7.9	0.93
45	stress B	Stress	9.37	1.36	21.84	3.34	7.1	0.93
4	stress A	Stress	9.57	3.59	3.49	4.53	7.8	0.96
3	stress A	Stress	9.68	2.19	5.70	5.81	8.4	0.98
31	stress B	Stress	9.83	0.23	5.06	5.06	8.1	0.95
71	stress C	Stress	10.12	2.08	5.70	3.37	7.1	0.94
11	stress A	Stress	10.17	2.47	20.11	4.16	7.6	0.95
69	stress C	Stress	10.61	0.8	25.13	3.76	7.2	1.01
39	stress B	Stress	10.75	1.75	29.61	4.96	8.4	0.84
5	stress A	Stress	10.97	2.13	8.98	5.57	8.2	1.01

12	stress A	Stress	11.27	1.98	12.98	4.42	7.7	0.97
14	stress A	Stress	11.67	1.09	25.22	5.30	8.5	0.86
70	stress C	Stress	12.35	3.55	15.81	3.37	6.6	1.17
64	stress C	Stress	12.90	3.36	36.08	4.95	7.8	1.04
13	stress A	Stress	13.57	1.08	58.83	3.81	7.5	0.90
76	stress C	Stress	14.40	2.14	8.32	3.91	7.8	0.82
65	stress C	Stress	15.30	3.52	15.31	4.89	8.1	0.92
40	stress B	Stress	17.21	7.9	51.67	3.39	7.2	0.91
46	stress B	Stress	17.62	1.95	17.71	4.14	7.5	0.98
72	stress C	Stress	17.67	4.22	12.18	3.96	7.5	0.94
75	stress C	Stress	19.75	4.75	16.39	5.65	8.5	0.92
50	stress B	Stress	21.26	9.45	46.36	3.38	7.3	0.87
41	stress B	Stress	22.15	6.29	63.40	6.23	8.4	1.05
77	stress C	Stress	25.39	1.31	12.16	3.51	7.4	0.87
47	stress B	Stress	28.51	2.6	39.36	3.83	7.4	0.94
35	stress B	Stress	28.70	1.01	65.81	4.30	8.0	0.84
17	stress A	Stress	29.10	4.87	33.42	5.23	8.4	0.88
